# Correction: Microbial diversity of coastal microbial mats formations in karstic habitats from the Yucatan Peninsula, Mexico

**DOI:** 10.1371/journal.pone.0349296

**Published:** 2026-05-12

**Authors:** Santiago Cadena, Claudia Teutli-Hernández, Jorge A. Herrera-Silveira, M. Leopoldina Aguirre-Macedo, Luisa I. Falcón, Brad M. Bebout, José Q. García-Maldonado

There are errors in the Funding statement. The correct Funding statement is as follows: This study was funded by Consejo Nacional de Ciencias, Humanidades y Tecnologías (CONAHCYT) project CF-2019–848287 awarded to JQGM. https://conahcyt.mx/. In addition, sampling was performed with support of Programa de Apoyo a Proyectos de Investigación e Innovación Tecnológica (PAPIIT-UNAM) Project IN216219. The funders had no role in study design, data collection and analysis, decision to publish, or preparation of the manuscript.

## References

[1] Cadena S, Teutli-Hernández C, Herrera-Silveira JA, Aguirre-Macedo ML, Falcón LI, Bebout BM, et al. Microbial diversity of coastal microbial mats formations in karstic habitats from the Yucatan Peninsula, Mexico. PLoS One. 2025;20(6):e0325200. doi: 10.1371/journal.pone.0325200 40460360 PMC12133189

