# Supplementary material for: Microbial diversity of coastal microbial mats formations in karstic habitats from the Yucatan Peninsula, Mexico
Source: PLoS One. 2025 Jun 3;20(6):e0325200. doi: 10.1371/journal.pone.0325200 (PMC12133189; doi:10.1371/journal.pone.0325200)
Supplement: S1 Table — Comparative table of the mineral composition of studied microbial mats from the Yucatan Peninsula. (x) presence, (-) absence. (DOCX) [file pone.0325200.s001.docx]

**Supplementary Table S1**

Table 1: Comparative table of the mineral composition of studied microbial mats from the Yucatan Peninsula. (x) presence, (-) absence.

| Microbial mats /minerals | Ankerite | Aragonite | Beidellite | Calcite | Calcite-magnesium | Gypsum | Halite | Hexahedrite | Quartz |
| --- | --- | --- | --- | --- | --- | --- | --- | --- | --- |
| Lift-off mats- Sisal | - | x | - | x | x | - | x | - | - |
| Flat - Progreso | - | x | x | x | x |  | x | - | x |
| Pustular - Progreso | x | x | - | x | x | x | x | - | - |
| Flat - Ría Lagartos | - | x | - | x | - | x | x | x | - |
