# Supplementary material for: Microbial diversity of coastal microbial mats formations in karstic habitats from the Yucatan Peninsula, Mexico
Source: PLoS One. 2025 Jun 3;20(6):e0325200. doi: 10.1371/journal.pone.0325200 (PMC12133189; doi:10.1371/journal.pone.0325200)
Supplement: S3 Table — Statistical analysis on the UniFrac distance matrix, using 16S rRNA gene sequences from microbial mats and corresponding environmental data. Asterisks denote statistically significant data. (DOCX) [file pone.0325200.s003.docx]

**Supplementary Table S2**

Table 2: Statistical analysis on the UniFrac distance matrix, using 16S rRNA gene sequences from microbial mats and corresponding environmental data. Asterisks denote statistically significant data.

| **Variable** | **P** | **R^2^** |
| --- | --- | --- |
| **Salinity** | 0.001* | 0.17 |
| **Temperature** | 0.001* | 0.13 |
| **pH** | 0.013 | 0.05 |
| **Redox potential** | 0.001* | 0.10 |
