# Supplementary figures and images for: Microbial diversity of coastal microbial mats formations in karstic habitats from the Yucatan Peninsula, Mexico
Source: PLoS One. 2025 Jun 3;20(6):e0325200. doi: 10.1371/journal.pone.0325200 (PMC12133189; doi:10.1371/journal.pone.0325200)

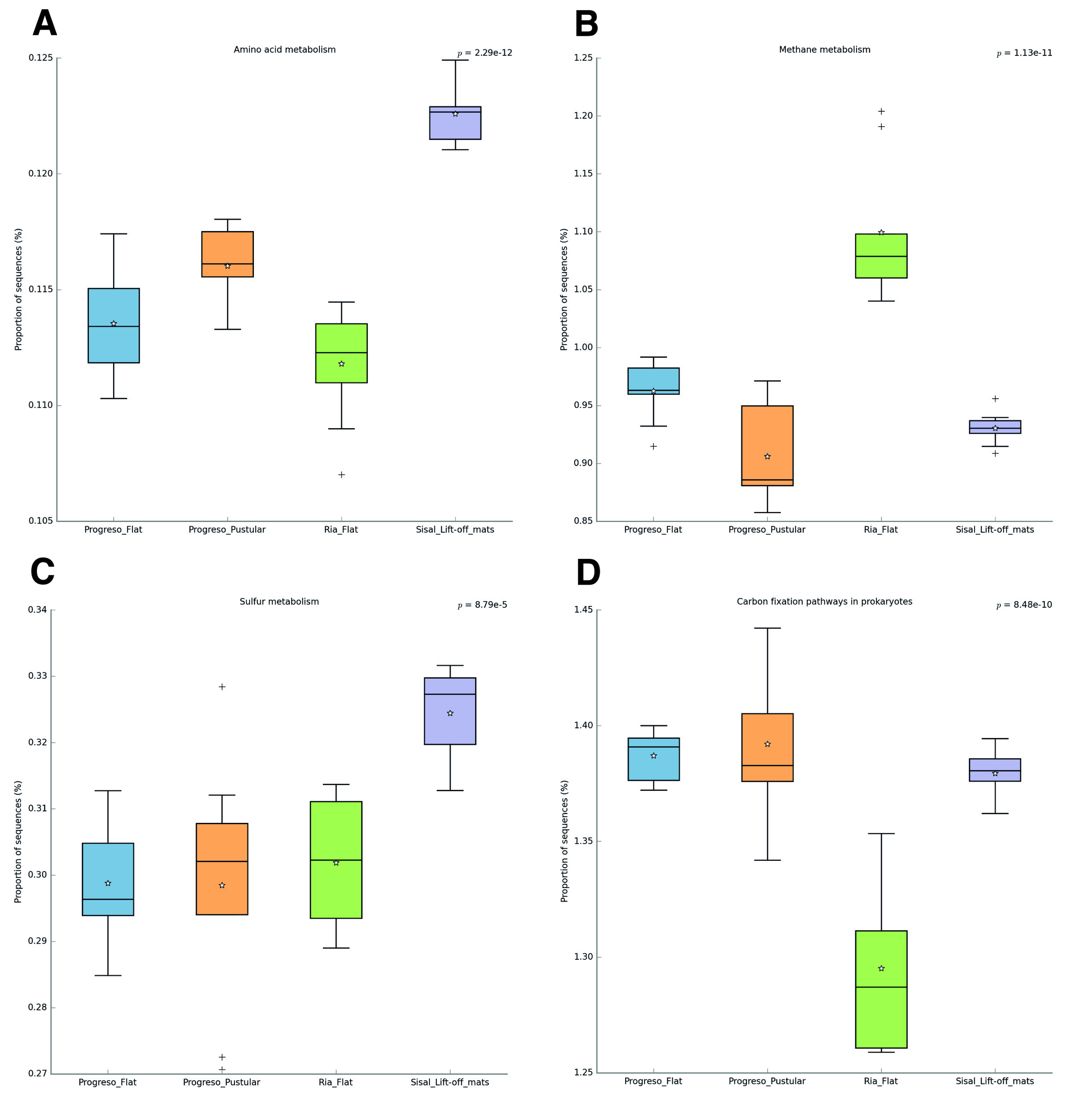

Supplement: S1 Fig — Comparative analysis of metabolic pathways across microbial mat types, highlighting amino acid, methane, sulfur, and carbon fixation metabolisms. (TIF) [file pone.0325200.s006.tif]
