# Supplementary material for: Microbial diversity of coastal microbial mats formations in karstic habitats from the Yucatan Peninsula, Mexico
Source: PLoS One. 2025 Jun 3;20(6):e0325200. doi: 10.1371/journal.pone.0325200 (PMC12133189; doi:10.1371/journal.pone.0325200)
Supplement: Attachment — Submitted filename: PONE-D-24-48408.pdf [file pone.0325200.s007.pdf]

PLOS ONE

Microbial diversity of coastal microbial mats formations in karstic habitats from the Yucatan Peninsula, Mexico

--Manuscript Draft--

**Manuscript Number:**	PONE-D-24-48408
**Article Type:**	Research Article
**Full Title:**	Microbial diversity of coastal microbial mats formations in karstic habitats from the Yucatan Peninsula, Mexico
**Short Title:**	Microbial mats from the Yucatan Peninsula
**Corresponding Author:**	José Q. García-Maldonado, Ph.D<br>Centro de Investigacion y de Estudios Avanzados Unidad Merida<br>Merida, Yucatan MEXICO
**Keywords:**	microbial mats; karstic ecosystem; 16S rRNA gene amplicon sequencing; Yucatan Peninsula
**Abstract:**	In this study, we report for the first time an exploration of the physicochemical characteristics and the prokaryotic diversity of three different types of microbial mats from karstic habitats located in Sisal, Progreso and Ría Lagartos, in the Yucatan Peninsula, Mexico. Our results showed that lift-off mats were found in the lower salinity (2.2%) area (Sisal), while flat and pustular mats were detected in hypersaline (6-9%) sites (Progreso and Ría Lagartos). Notably, some of these microbial mat structures were in close proximity to mangrove forest ecosystems with both degraded and restored regimes. XRD analysis revealed different mineral compositions of the mats; however, aragonite, calcite, and halite were commonly found in all samples studied. High-throughput sequencing of the 16S rRNA gene identified differences in microbial communities across the different mat types, and statistical analyses revealed that salinity, redox potential, and temperature were significant factors in explaining the variance of the prokaryotic assemblages. Microbial groups identified in this study include those known to be important in the biogeochemical cycling of key elements, such as carbon, nitrogen, and sulfur. Interestingly, the community composition of flat and pustular mats from Progreso was similar, with Bacteroidia, Anaerolineae, and Phycisphaerae being the most abundant microbial groups in flat mats, and Bacteroidia, Anaerolineae, and Alphaproteobacteria dominating pustular mats. By contrast, flat mats from Ría Lagartos were dominated by Halobacteria, Cyanobacteria and Bacteroidota, while lift-off mats from Sisal were dominated by Bacteroidia, Gammaproteobacteria and Cyanobacteria. This work contributes to the understanding of the distribution, physicochemical characteristics and microbial diversity of coastal microbial mats, providing valuable new insights into microbial mats that develop in karstic ecosystems. This information is relevant to ongoing and future efforts to manage and preserve coastal ecosystems in the Yucatan Peninsula.
**Order of Authors:**	Santiago Cadena
	Claudia Teutli-Hernández
	Jorge A. Herrera-Silveira
	M. Leopoldina Aguirre-Macedo
	Luisa I. Falcón
	Brad M. Bebout
	José Q. García-Maldonado, Ph.D
**Additional Information:**	
**Question**	**Response**
**Financial Disclosure**<br><br>Enter a financial disclosure statement that	Yes

Powered by Editorial Manager® and ProduXion Manager® from Aries Systems Corporation

describes the sources of funding for the work included in this submission. Review the submission guidelines for detailed requirements. View published research articles from *PLOS ONE* for specific examples.<br><br>This statement is required for submission and **will appear in the published article** if the submission is accepted. Please make sure it is accurate.<br><br>**Funded studies**<br>Enter a statement with the following details:<br>• Initials of the authors who received each award<br>• Grant numbers awarded to each author<br>• The full name of each funder<br>• URL of each funder website<br>• Did the sponsors or funders play any role in the study design, data collection and analysis, decision to publish, or preparation of the manuscript?<br><br>Did you receive funding for this work?	
Please add funding details.<br>as follow-up to "**Financial Disclosure**<br><br>Enter a financial disclosure statement that describes the sources of funding for the work included in this submission. Review the submission guidelines for detailed requirements. View published research articles from *PLOS ONE* for specific examples.<br><br>This statement is required for submission and **will appear in the published article** if the submission is accepted. Please make sure it is accurate.	This study was funded by Consejo Nacional de Ciencias, Humanidades y Tecnologías (CONAHCYT) project CF-2019-848287 awarded to JQGM.<br>https://conahcyt.mx/<br>. In addition, sampling was performed thanks to Programa de Apoyo a Proyectos de Investigación e Innovación Tecnológica (PAPIIT-UNAM) IN216219 awarded to CTH.<br>https://dgapa.unam.mx/

**Funded studies**<br>Enter a statement with the following details:<br>• Initials of the authors who received each award<br>• Grant numbers awarded to each author<br>• The full name of each funder<br>• URL of each funder website<br>• Did the sponsors or funders play any role in the study design, data collection and analysis, decision to publish, or preparation of the manuscript?<br><br>Did you receive funding for this work?"	
Please select the country of your main research funder (please select carefully as in some cases this is used in fee calculation).<br>as follow-up to "**Financial Disclosure**<br><br>Enter a financial disclosure statement that describes the sources of funding for the work included in this submission. Review the submission guidelines for detailed requirements. View published research articles from *PLOS ONE* for specific examples.<br><br>This statement is required for submission and **will appear in the published article** if the submission is accepted. Please make sure it is accurate.<br><br>**Funded studies**<br>Enter a statement with the following details:<br>• Initials of the authors who received each award<br>• Grant numbers awarded to each author<br>• The full name of each funder<br>• URL of each funder website<br>• Did the sponsors or funders play any role in the study design, data collection and analysis, decision to publish, or preparation of the manuscript?<br><br>Did you receive funding for this work?"	MEXICO - MX
**Competing Interests**	The authors have declared that no competing interests exist.

Use the instructions below to enter a competing interest statement for this submission. On behalf of all authors, disclose any competing interests that could be perceived to bias this work—acknowledging all financial support and any other relevant financial or non-financial competing interests.<br><br>This statement is required for submission and **will appear in the published article** if the submission is accepted. Please make sure it is accurate and that any funding sources listed in your Funding Information later in the submission form are also declared in your Financial Disclosure statement.<br><br>View published research articles from *PLOS ONE* for specific examples.<br><br>**NO authors have competing interests**<br><br>Enter: *The authors have declared that no competing interests exist.*<br><br>**Authors with competing interests**<br><br>Enter competing interest details beginning with this statement:<br><br>*I have read the journal's policy and the authors of this manuscript have the following competing interests: [insert competing interests here]*<br><br>* typeset	
**Ethics Statement**<br><br>Enter an ethics statement for this submission. This statement is required if the study involved:<br><br>• Human participants<br>• Human specimens or tissue<br>• Vertebrate animals or cephalopods<br>• Vertebrate embryos or tissues	Field Research.<br>Document: SPARN/DGVS/04857/23<br>Subsecretaria de Politica Ambiental y Recursos Naturales, Direccion General de Vida Silvestre

• Field research<br><br>Write "N/A" if the submission does not require an ethics statement.<br><br>General guidance is provided below. Consult the submission guidelines for detailed instructions. **Make sure that all information entered here is included in the Methods section of the manuscript.**	

Powered by Editorial Manager® and ProduXion Manager® from Aries Systems Corporation

**Format for specific study types**<br><br>**Human Subject Research (involving human participants and/or tissue)**<br>• Give the name of the institutional review board or ethics committee that approved the study<br>• Include the approval number and/or a statement indicating approval of this research<br>• Indicate the form of consent obtained (written/oral) or the reason that consent was not obtained (e.g. the data were analyzed anonymously)<br><br>**Animal Research (involving vertebrate animals, embryos or tissues)**<br>• Provide the name of the Institutional Animal Care and Use Committee (IACUC) or other relevant ethics board that reviewed the study protocol, and indicate whether they approved this research or granted a formal waiver of ethical approval<br>• Include an approval number if one was obtained<br>• If the study involved *non-human primates*, add *additional details* about animal welfare and steps taken to ameliorate suffering<br>• If anesthesia, euthanasia, or any kind of animal sacrifice is part of the study, include briefly which substances and/or methods were applied<br><br>**Field Research**<br><br>Include the following details if this study involves the collection of plant, animal, or other materials from a natural setting:<br>• Field permit number<br>• Name of the institution or relevant body that granted permission	
**Data Availability**<br><br>Authors are required to make all data underlying the findings described fully available, without restriction, and from the time of publication. PLOS allows rare exceptions to address legal and ethical concerns. See the PLOS Data Policy and FAQ for detailed information.	Yes - all data are fully available without restriction

A Data Availability Statement describing where the data can be found is required at submission. Your answers to this question constitute the Data Availability Statement and **will be published in the article**, if accepted.<br><br>**Important:** Stating 'data available on request from the author' is not sufficient. If your data are only available upon request, select 'No' for the first question and explain your exceptional situation in the text box.<br><br>Do the authors confirm that all data underlying the findings described in their manuscript are fully available without restriction?	
**Describe where the data may be found in full sentences. If you are copying our sample text, replace any instances of XXX with the appropriate details.**<br><br>• If the data are **held or will be held in a public repository**, include URLs, accession numbers or DOIs. If this information will only be available after acceptance, indicate this by ticking the box below. For example: *All XXX files are available from the XXX database (accession number(s) XXX, XXX.*).<br>• If the data are all contained **within the manuscript and/or Supporting Information files**, enter the following: *All relevant data are within the manuscript and its Supporting Information files.*<br>• If neither of these applies but you are able to provide **details of access elsewhere**, with or without limitations, please do so. For example:<br><br>*Data cannot be shared publicly because of [XXX]. Data are available from the XXX Institutional Data Access / Ethics Committee (contact via XXX) for researchers who meet the criteria for access to confidential data.*<br><br>*The data underlying the results presented in the study are available from (include the name of the third party*	The 16S raw sequences generated in this study were deposited in the NCBI Sequence Read Archive under the BioProject PRJNA971606.

and contact information or URL).<br>• This text is appropriate if the data are owned by a third party and authors do not have permission to share the data.<br><br>* typeset	
Additional data availability information:	

Powered by Editorial Manager® and ProduXion Manager® from Aries Systems Corporation

Manuscript

Click here to access/download;Manuscript;MS_Yucamats_2024.docx

Microbial diversity of coastal microbial mats formations in karstic habitats from the Yucatan Peninsula, Mexico

Santiago Cadena[1], Claudia Teutli-Hernández[2], Jorge A. Herrera-Silveira[3], M. Leopoldina Aguirre-Macedo[3], Luisa I. Falcón[4], Brad M. Bebout[5], José Q. García-Maldonado[3*]

[1]Centro de Investigaciones Químicas, Universidad Autónoma del Estado de Morelos, Cuernavaca, Morelos, Mexico

[2]Escuela Nacional de Estudios Superiores Mérida, Universidad Nacional Autónoma de México, Ucú, Yucatán, Mexico

[3]Centro de Investigación y de Estudios Avanzados Del Instituto Politécnico Nacional, Departamento de Recursos Del Mar, Unidad Mérida, Yucatan, Mexico

[4]Departmento de Ecología Evolutiva, Instituto de Ecología, Unidad Mérida, Universidad Nacional Autónoma de México, Mexico

[5]Exobiology Branch, Ames Research Center, National Aeronautics and Space Administration, Moffett Field, CA, USA

*corresponding author

E-mail: jose.garcia@cinvestav.mx

Abstract

In this study, we report for the first time an exploration of the physicochemical characteristics and the prokaryotic diversity of three different types of microbial mats from karstic habitats located in Sisal, Progreso and Ría Lagartos, in the Yucatan Peninsula, Mexico. Our results showed that lift-off mats were found in the lower salinity (2.2%) area (Sisal), while flat and pustular mats were detected in hypersaline (6-9%) sites (Progreso and Ría Lagartos). Notably, some of these microbial mat structures were in close proximity to mangrove forest ecosystems with both degraded and restored regimes. XRD analysis revealed different mineral compositions of the mats; however, aragonite, calcite, and halite were commonly found in all samples studied. High-throughput sequencing of the 16S rRNA gene identified differences in microbial communities across the different mat types, and statistical analyses revealed that salinity, redox potential, and temperature were significant factors in explaining the variance of the prokaryotic assemblages. Microbial groups identified in this study include those known to be important in the biogeochemical cycling of key elements, such as carbon, nitrogen, and sulfur. Interestingly, the community composition of flat and pustular mats from Progreso was similar, with Bacteroidia, Anaerolineae, and Phycisphaerae being the most abundant microbial groups in flat mats, and Bacteroidia, Anaerolineae, and Alphaproteobacteria dominating pustular mats. By contrast, flat mats from Ría Lagartos were dominated by Halobacteria, Cyanobacteria and Bacteroidota, while lift-off mats from Sisal were dominated by Bacteroidia, Gammaproteobacteria and Cyanobacteria. This work contributes to the understanding of the distribution, physicochemical characteristics and microbial diversity of coastal microbial mats, providing valuable new insights into microbial mats that develop in karstic ecosystems. This information is relevant to ongoing and future efforts to manage and preserve coastal ecosystems in the Yucatan Peninsula.

Keywords: microbial mats, karstic ecosystem, 16S rRNA gene amplicon sequencing, Yucatan Peninsula

Introduction

Microbial mats are intricate associations of multiple functional groups of microbes that grow on surfaces such as sediments, rocks and sand [1], that can be found in diverse aquatic environments including hot springs, hypersaline ponds, alkaline lakes, dry and hot deserts, and coastal intertidal areas [2]. Mats typically flourish in zones with minimal competition from plants and grazing organisms [3], possibly due to the adverse effects of nutrient-poor conditions, and only periodic inundation, leading to desiccation and significant fluctuations in temperature and salinity [1].

Microbial mats can be differentiated based on their complex morphologies. These morphologies include the typical flat mat, but also include pustular, gelatinous, tufted, reticulate, and blister mats, among others [4-6]. Mat formation is shaped by diverse abiotic factors, such as water availability, sediment influx, wind intensity and type of substrate [3-4, 7]. In addition, it is known that different mat morphologies contain diverse microbial communities [8-9]. Up to now, the most examined types of coastal mats are located in Australia. Flat microbial mats from Shark Bay have been reported to be dominated by archaeal communities from Parvarchaeota and Thermoplasmata, while pustular mats are dominated by Halobacteria [10]. Pustular mats from Hamelin Pool are dominated by the Cyanobacteria *Entophysalis*, compared to flat mats that usually contain *Coleofasciculus* [11].

Proteobacteria, Bacteroidetes and Planctomycetes are the most common taxa in pustular mats from Carbla Beach, where Cyanobacteria from Pseudophormidiales and Phormidesmiales are responsible for the production of sulfated extracellular polymeric substances [9]. D'Agostino and colleagues [12] investigated flat, pustular and tufted mats from Hamelin Pool indicating that the shape of the mat helps to protect microbial communities from ultraviolet radiation and environmental pressures. Eukaryotic diversity in these two mat types is also different; flat mats can contain nematodes, fungi and diatoms while pustular mats harbor fungi, diatoms, tardigrades and microalgae [8]. Although diverse studies have investigated the differences in coastal microbial mats, karstic microbial mats have received comparatively less attention than other ecosystems.

Karst is defined as a special type of landscape developed on soluble rocks, such as limestone, marble, dolomite, and gypsum [13]. The Yucatan Peninsula (YP) is one of the most extensive karst systems known on the planet, encompassing a limestone platform of 165,000 km^2 [14]. The climatic conditions of the YP are divided into three seasons: dry (March-June), rainy (July-October), and north-winds (November-February). Due to its coastal features, the YP provides ideal conditions for the formation of shallow, swampy, brackish-to-saline estuaries along the shoreline [15]. Furthermore, the hydrological properties of the YP's coast dictate that evaporation rates are significantly higher than water contributions, leading to the development of numerous solar salterns in the coastal area [16]. Favorable environmental conditions for mat formation led to widespread occurrence of these microbial communities, however, information on coastal microbial mats in this region is limited. Therefore, this study aims to explore and characterize, for the first time, the prokaryotic community structure of diverse types of mats from different locations along the YP, using 16S rRNA gene sequencing.

Materials and methods

Sampling and physicochemical characterization

Microbial mats were collected in May 2019 from three coastal zones in Yucatan, Mexico, specifically from Sisal, Progreso, and Ría Lagartos (Fig 1). Notably, some of the microbial mats were found in close proximity to mangrove forests which are degraded, as well as some which have been managed and restored from a degraded condition. At each locality, triplicate surficial mat cores measuring 8 cm in width by 8 cm in length and 3-5 cm in depth were collected. Subsequently, from each of the previously obtained cores, three sub-cores from the upper first centimeter of the mats (1 cm × 1 cm) was taken to obtain 9 representative samples per locality. The sub-cores were immediately stored in liquid nitrogen for sequencing. Interstitial water from each locality was obtained using a 50 ml syringe coupled to an acrylic tube sealed at the bottom but containing a small hole. The tube was inserted into the mats to 30 centimeters depth and the water was extracted by suction with the syringe to obtain 40 ml of interstitial water. This device allows the sampling of interstitial water with low or null exposure to atmospheric air [17]. Physicochemical variables including salinity, temperature, pH, and redox potential were measured immediately from interstitial water using a portable multi-parameter analyzer. Each environmental variable was measured 9 times at each site, except for Ría Lagartos, where technical difficulties on site only allowed for a single data point to be collected for each variable.

Fig 1. Sampling sites of microbial mats from the Yucatan Peninsula. Different types of coastal microbial mats were documented. Lift-off mats from Sisal (B), Pustular (C) and flat (D) microbial mats from Progreso, and flat mats from Ría Lagartos (E).

Mineralogical analysis by XRD

Samples of microbial mats were dried at room temperature and then finely macerated (<20 um) and placed into a muffle for 12 hours at 550°C to calcinate organic materials. To determine the mineral composition of the microbial mats, samples were subjected to X-ray diffraction (XRD) analysis using a Bruker D-8 Advance (IUC, Indore) diffractometer with a Cu tube (kα: 1.5406 Å) operated at 30 mÅ and 40 kV, at CINVESTAV-Mérida.

DNA extraction and amplicon library construction for sequencing

DNA of collected microbial mats from each locality (n=9) was extracted from 0.25 g of microbial mat sample using the PowerSoil DNA Isolation Kit (Mo Bio Laboratories, Carlsbad, CA, USA) following the conventional instructions, with cell lysis carried out using a TissueLyser LT (Qiagen, Hilden, Germany). To monitor potential contamination, a blank (a column with no sample supplied) was processed alongside the extractions. DNA quality was assessed by running samples on a 1% agarose gel.

To amplify 16S rRNA gene fragments from Bacteria and Archaea, universal primer sets 515F-Y and 926R were used, which cover the V4-V6 regions [17]. The primer characteristics and thermocycling conditions were detailed by Needham and colleagues [18]. PCR reactions (20 µl) were performed using 2 µl of DNA (5 ng/µl), 0.5 µl of each primer (10 µM), and 10 µl of 2× Phusion High-Fidelity MasterMix (Thermo Scientific, Waltham, MA, USA). PCR fragments were first purified using magnetic beads (AMPure XP) from Beckman Coulter Genomics (Brea, CA, USA), and subsequently, amplicons were indexed using the Nextera XT Index kit version 2 (Illumina, San Diego, CA, USA), following the Illumina 16S Metagenomic Sequencing Library Preparation protocol. After indexing, barcoded fragments were purified a second time and quantified using the Qubit 3.0 fluorometer from Life Technologies (Malaysia). The correct size of PCR products was confirmed on an Advanced QIAxcel from QIAGEN (USA). Barcoded PCR-amplicons were diluted in 10 mM Tris (pH 8.5) and pooled in equimolar concentrations (9 pM). Finally, paired-end sequencing was performed on the MiSeq platform (Illumina, San Diego, CA, USA) with a MiSeq Reagent Kit V3 (600 cycles), using the Aquatic Pathology Laboratory at CINVESTAV-Mérida. All sequence data is publicly available in the NCBI Sequence Read Archive under the BioProject PRJNA971606.

The QIIME2 (2017.11) pipeline [19] was utilized to analyze demultiplexed reads. To denoise and resolve amplicon sequence variants (ASV), the DADA2 plugin was employed, and chimeras were eliminated using the "consensus" method [20]. Representative ASVs were assigned using the SILVA small subunit ribosomal RNAs (16S) v138 database as a reference through the V-SEARCH plugin [21]. To build a phylogenetic tree, representative reads were aligned with the MAFFT algorithm [22] and filtered using fasttree [23]. The data were

normalized by sub-sampling to the lowest read count per sample (10,700). R environment was used for graphic visualization and statistical analysis, with phyloseq [24] and ggplot2 [25] libraries. Additionally, alpha diversity (observed ASVs, Shannon and Simpson indexes) and beta diversity from samples were calculated. One-way ANOVA was performed to determine the significance of the differences between alpha diversity measurements, followed by a post hoc Tukey's test when significant differences were identified. Furthermore, in order to characterize the most representative ASVs of each type of microbial mat, we used a Linear Discriminant Analysis (LDA) Effect Size (LEfSe) [26], employing an LDA threshold >2, coupled with internal Kruskal-Wallis and Wilcoxon tests, ensuring significance at a p-value <0.05, performed in the Galaxy web application [27].

Results

Physicochemical characteristics

Different types of microbial mats were found, denominated as lift-off mats, pustular, and flat mats. Salinity measurements taken at the study sites ranged from 2.2% to 9.8%, with the lowest values found at Sisal and the highest at Ría Lagartos. The average temperature of the interstitial water varied from 31 to 37° C. pH values were similar across all sites, with an average of 7.3 ± 0.2. The redox potential measurements ranged from −177 to −299 mV. The physicochemical properties of the microbial mats that were sampled are provided in Table 1.

Table 1. Physicochemical characterization.

Locality	Macrostructure	Type of associated mangrove	Salinity (%)	Temperature (°C)	pH	Redox potential (mV)
**Sisal**	Lift-off mats	Degraded	2.2 (±0.22)	31.4 (±0.72)	7.4 (±0.07)	-299.6 (±20.08)
**Progreso**	Pustular mat	Restored	6.0 (±0.36)	34.6 (±1.29)	7.2 (±0.06)	-271.1 (±21.07)
**Progreso**	Flat mat	Degraded	8.8 (±0.54)	32.4 (±1.63)	7.4 (±0.11)	-177.7 (±60.33)
**Ría Lagartos**	Flat mat	No mangrove	9.8	37.3	7.2	-207.0

Sampled sites and type of macrostructure.

Nine different minerals were detected by XRD analysis of the microbial mats (Fig 2). Calcite and halite were found to be present in all the studied microbial mats, including the lift-off mats, flat, and pustular mats. In addition, beidellite and quartz were only found in the flat mats from Progreso, highlighting the differences in mineral

composition across the different mat types. A more detailed comparison of the mineral composition of the microbial mats is shown in Supplementary Table S1.

Fig 2. X-ray diffraction patterns of microbial mat samples collected from different locations. Lift-off mats from Sisal (A); flat (B) and pustular (C) mats from Progreso and flat mats from Ría Lagartos (D).

Microbial community structure analysis

Samples of all microbial mats were sequenced including lift-off mats from Sisal, pustular and flat mats from Progreso, and flat mats from Ría Lagartos. Sequencing of 16S rRNA gene obtained 870,175 raw reads from studied mats, yielding a total of 751,471 high quality sequences. Quality filtering is shown in Supplementary Table S2. The observed amplicon sequence variants (ASVs) ranged from 729 to 1081, and the Shannon diversity index ranged from 5.76 to 6.23, while the Simpson index varied between 0.992 and 0.996 (Figure 3A). ANOVA showed significant differences in alpha diversity measurements (observed ASVs, Shannon and Simpson indexes) among the sampling locations ($p < 0.001$). Tukey's test further revealed that Ría Lagartos had significantly lower diversity compared to the other sites ($p < 0.001$). Principal Coordinates Analysis (PCoA) based on the weighted and unweighted UniFrac distances demonstrated that the microbial community structure of the mats was distinct among the different sampled sites (Fig 3B and 3C).

Fig 3. Alpha and beta diversity of microbial mats samples. Observed ASVs, Shannon and Simpson indexes (A). Localities that are significantly different are denoted with different letters (a, b or c) (one-way ANOVA, Tukey's test, $p < 0.001$). PCoA calculated on the unweighted (B) and weighted (C) UniFrac metrics, based on 16S rRNA gene amplicon sequences.

The results of the PERMANOVA analysis between environmental data and relative abundance of ASVs indicated that salinity, temperature, and redox potential influenced microbial community structure of studied microbial mats (Table 2). Specifically, salinity was found to explain 17% of the variance, while temperature and redox potential explained 13% and 10%, respectively, indicating the importance of these environmental variables in shaping the composition of microbial communities in these mats.

Table 2: PERMANOVA analysis.

Variable	P	R^2
**Salinity**	0.001*	0.17
**Temperature**	0.001*	0.13
**pH**	0.013	0.05
**Redox potential**	0.001*	0.10

Statistical analysis on the UniFrac distance matrix, using 16S rRNA gene sequences from microbial mats and corresponding environmental data.

Asterisks denote statistically significant data.

Microbial community composition

The archaeal communities detected in the microbial mat samples were affiliated with nine phyla, including Halobacterota, Nanoarchaeota, Asgardarchaeota, and Thermoplasmatota as the most represented. Archaeal communities in microbial mats from Sisal and Progreso ranged from 3% to 7% of the total ASVs. In contrast, mats from Ría Lagartos contained between 20% to 35% of Halobacterota.

The bacterial community in the microbial mats was highly diverse, covering 39 phyla, but was dominated by Bacteroidetes, Proteobacteria, Chloroflexi, Planctomycetota, and Cyanobacteria (Fig 4). Bacteroidetes (40-13%) was composed of several classes, including Bacteroidia (37-5%), Rhodothermia (17-1%), and Ignavibacteria (2-0.2%). Proteobacteria (23-7%) contained members from Gammaproteobacteria (19-3%) and Alphaproteobacteria (3-6%) classes, while Chloroflexi (28-4%) included Anaerolineae (21-3%) and Chloroflexia (7-1%). Planctomycetota (14-2%) was mainly represented by Phycisphaerae (9-1%). Finally, Cyanobacteria accounted for (19-5%) of diversity.

The PCoA analysis indicated that the community composition of flat and pustular mats from Progreso were similar to each other (Figure 3B and 3C). In flat mats from Progreso, Bacteroidetes (Bacteroidia, 22-17%), Chloroflexi (Anaerolineae, 19-12%), and Planctomycetes (Phycisphaerae, 8-9%) were the most abundant microbial groups. In comparison, pustular mats from the same location were dominated by Bacteroidetes (Bacteroidia, 2-13%), Chloroflexi (Anaerolineae, 21-14%), and Proteobacteria (Alphaproteobacteria, 4-12%). In contrast, flat mats from Ría Lagartos were dominated by Halobacterota (Halobacteria, 35-17%), Cyanobacteria (19-6%), and Bacteroidota (Rhodothermia, 17-7%). Lift-off mats from Sisal were found to contain Bacteroidetes (Bacteroidia, 37-25%), Proteobacteria (Gammaproteobacteria, 16-19%), and Cyanobacteria (7-5%) as the most abundant microbial groups (Fig. 4). Detailed information on the microbial diversity detected on mats at the ASV level is presented in Supplementary Table S3.

Fig 4. Relative abundance of taxonomic groups of microbes observed . Stacked bars at phylum (A) and class (B) level from studied microbial mats. Clades represented <1% were grouped in "Others".

LefSe analysis identified the most representative ASVs from each studied microbial mat. Candidatus Chlorothrix, Phycisphaeraceae, AKAU3564 sediment group, A4b, and Sb_5 were the most representative taxa in flat mats from Progreso, while pustular mats from Progreso were characterized by SBR1031, Phaeodactylibacter, Ardenticatenales, Zixivacteria, and Spirochaet. Flat mats from Ría displayed *Salinibacter*, *Balneolaceae, Coleofasciculus_PCC_7420*, Haloferacaceae, and Halomicrobiaceae, and lift-off mats from Sisal had *Thioalkalovibrio*, *Robiginitalea*, *Lentimicrobiaceae*, VC2_1 Bac22, and *Thiohalocapsa* as the most represented taxa. Figure 5 shows the top 10 taxa with the highest LDA scores.

Fig 5. LefSe analysis based on retrieved 16S rRNA sequences from microbial mat samples. The top 10 organisms with the highest LDA scores are shown, indicating their potential as biomarkers for each mat type.

Discussion

Previous studies of the YP have identified microbial mats and microbialites in various aquatic systems such as Laguna Bacalar [28], freshwater cenotes [29-30], and the Chicxulub crater [31]. The current study provides novel insights by reporting the occurrence of microbial mats along the coastline of YP, in three distinct locations: Sisal, Progreso, and Ría Lagartos, exhibiting unique physicochemical characteristics and harboring different types of microbial mats.

Physicochemical characteristics of mats and prokaryotic community structure

Benthic microbial mats without much vertical relief, are commonly known as smooth mats [1-6]. This type of macrostructure was observed in Progreso, close to degraded mangroves, and at Ría Lagartos in the absence of mangrove ecosystems. In contrast, pustular mats have a tufted structure, which distinguishes them, albeit somewhat subjectively, from flat mats [8]. Pustular mats were found at Progreso, associated with mangrove ecosystems with hydrological rehabilitation, that consist in the construction of canals to facilitate water input [7-8]. It has been suggested that as mangroves recover, there is a progression from smooth to pustular mats, which can be visualized as columnar growth [4,10]. Additionally, mat formations are influenced by a variety of environmental factors, such as sediment influx, substrate elevation, interstitial groundwater, waves or currents, hydroperiod, among others [5, 7]. Mats can even become separated from the benthic substrate. Physical disruption caused by temperature, gas production, or flooding can release the mat from the sediment, resulting in a floating

mass in water. Lift-off mats have been discovered in salt lakes [31], caves [32] and hot springs [33]. In this study, we report the presence of lift-off mats occurring at low salinity next to degraded mangroves in Sisal. A wide range of minerals associated with carbon, iron, and sulfur cycles were detected in flat, pustular, and lift-off mats (Fig 2). Halite and calcite were present in all studied microbial mats. These findings may be explained by the karstic characteristics of the region.

Salinity, temperature, and redox potential were the environmental variables that explained the variance in the taxonomic composition of the microbial mat communities. It is well known that those variables are key drivers of microbial populations in many environments. Salinity, for example, triggers osmotic stress that requires a complex metabolic specialization [35]. The negative correlation between salinity and alpha diversity was evident, with the highest salinity observed in Ría Lagartos resulting in significantly lower diversity. This pattern is consistent with previous studies in terrestrial and marine ecosystems. Temperature, through its overarching role in regulating rates of overall microbial activity and biogeochemical cycling also exerts a strong influence on microbial community composition [36]. Finally, redox potential both controls, and is controlled by, available electron donors and acceptors, and the presence or absence of microbes capable of using these electron donors and acceptors for metabolism. [37].

Mangrove ecosystems usually develop in the intertidal zone of tropical coastlines, where benthic microbial mats may also be found. In this work, we report microbial mat formations close to mangrove ecosystems in degraded conditions and with hydrological rehabilitation. Lugomela and Berman [38] documented the presence of microbial mats in mangrove forest from Zanzibar, Tanzania, quantifying higher nitrogen fixation rates in sediments covered with mats than in those without mats. Studies on benthic microbial mats from Twin Cays, Belize showed that mats are closely associated with mangroves, containing up to 30% by volume of live mangrove roots [39]. Additionally, mats can influence nitrogen availability to mangrove trees, providing nitrogen through nitrogen fixation in dwarf habitats, while in fringe and transition habitats, mats may compete with mangroves for nitrogen through denitrification [40]. Extensive microbial mat formation has been identified in mangrove sediments in the Manche-à-Eau lagoon, Guadeloupe French West Indies, influencing the carbon/nitrogen input in sediments, likely through mat-forming microorganisms that produce exopolysaccharides [41]. Undoubtedly, the contribution of microbial mats to biogeochemical cycling of mangrove ecosystems remains understudied. The participation of benthic microorganisms in biogeochemical cycling of mangrove habitats should be considered in conservation and rehabilitation/recovery efforts in mangrove ecosystems [42].

Prokaryotic diversity of flat, pustular, and lift-off mats

Microbial mats, which range in thickness from several millimeters to some centimeters, exhibit vertical layering that is composed of a few functional groups of microbes. The combined metabolic activities of these microbes create environmental microgradients, especially oxygen and hydrogen sulfide [3]. These mats primarily rely on autotrophy, using inorganic carbon to synthesize biomass through photosynthesis or chemosynthesis, followed by organic matter decomposition through aerobic and anaerobic degradation by fermenters and aerobes. This process can generate hydrogen sulfide and sulfate, which are used by green and purple sulfur bacteria as well as sulfate reducers, respectively [1-3].

The microbial community structure of both flat and pustular mats from Progreso was found to be relatively similar across all samples (Figs 3 and 4). These communities were mainly dominated by Bacteroidia, Chloroflexi, Planctomycetes, and Proteobacteria, which is consistent with observations from other microbial mats across the world, including those from Guerrero Negro [43], Elkhorn Slough [44] and Shark Bay [10], suggesting the relevance of these taxonomic groups occurring in mats independently of the geographic distance, which argues against functional redundancy, that states that other species can contribute similarly in ecosystem functioning [45]. Interestingly, flat mats from Ría Lagartos were dominated by the archaeal clade Halobacteria, Cyanobacteria, and Rhodothermia (Figure 4). These microbial communities are more similar to those reported in endoevaporites and salt crusts than those reported from microbial mats [46], which is consistent with the high salinity conditions of the site (close to 10%). Halobacterales constitute a clade found in hypersaline environments worldwide with autotrophic capabilities; Cyanobacteria is a clade that groups primary producers [3,43]. Additionally, lift-off mats from Sisal were dominated by Bacteroidia, Gammaproteobacteria, and Cyanobacteria (Figure 4). Information on lift-off mats from coastal environments is limited; Jhon and Paton [32] investigated lift-off mats at hypersaline conditions in coastal lakes of Western Australia, reporting different cyanobacteria, such as *Aphanothece*, *Oscillatoria*, and *Microcoleus*, using culture-dependent techniques. Our work provides important information on the dominant microbial taxa occurring in coastal lift-off mats, but temporal studies are required to understand the succession of lift-off mats, flat, and pustular mats on these sites.

The organisms with higher LDA value resulted from the LefSe analysis (employed to identify the most representative ASVs present in each of the studied microbial mats) indicated that: the flat mats from Progreso were characterized by Candidatus *Chlorothrix*, uncultured *Phycisphaeraceae* and AKAU3564. *Chlorothrix*, a, sulfide-dependent anoxygenic photoautotrophic bacterium, has been also observed in similar hypersaline microbial mats from Guerrero Negro [47]. Phycisphaeraceae, comprising facultative anaerobes, present in sediments performing anaerobic ammonium oxidation dependent on natural organic matter. The AKAU3564 clade is frequently found in marine sediments and is proposed to be associated with methane-oxidizing microorganisms [50]. The pustular mats from Progreso displayed SBR1031, uncultured *Phaeodactylibacter*, and uncultured Ardenticatenales. The environmental group SBR1031, also known as Aggregatilineales, belongs to the Chloroflexota phylum, commonly found in extreme environments [51]. Phaeodactylibacter has been reported as aerobic heterotrophs and fermenters in marine environments [52]. Members of Ardenticatenales have also been observed in coastal hot springs, being capable of using nitrate or ferric iron as electron acceptors [53]. The flat mats from Ría were dominated by *Salinibacter*, *Balneolaceae*, and *Coleofasciculus_PCC_7420*. *Salinibacter* is a fermentative extreme halophile bacterium repeatedly found in hypersaline ecosystems, particularly reported in solar salterns [54]. *Balneolaceae* is a family frequently identified in salt mines, solar salterns, or marine ecosystems, with heterotrophic metabolism involved in carbon degradation [55]. *Coleofasciculus* (formerly classified as *Microcoleus*) is a global cyanobacterium known as a mat builder due to its production of polymeric substances [3]. Finally, the lift-off mats from Sisal were represented by *Thioalkalovibrio*, *Robiginitalea*, and uncultured Lentimicrobiaceae. *Thioalkalovibrio* is a chemolithoautotrophic clade that reduces sulfur compounds [56]. *Robiginitalea* is a genus of bacteria known to be chemoheterotroph (obtain their energy from organic matter), and they have the ability to metabolize complex, high molecular weight compounds [57]. Members of Lentimicrobiaceae are considered to be strictly anaerobic fermenters, that are not capable of breaking down complex carbon compounds [58].

Conclusions

This work represents the first study in identifying and quantifying the prevalence of coastal microbial mats in the Yucatan Peninsula. Our findings shed new light on the distribution and ecological importance of these unique microbial communities, which may play a crucial role in shaping the coastal ecosystems of the region, since some of them were associated with mangrove forest ecosystems. Our findings showed a diverse array of microbial macrostructures, which have been previously classified as flat, lift-off mats, and pustular microbial mats. Each of these distinct types of mats exhibited unique physical and chemical properties, as well as significant different microbial communities. The dominant minerals identified in the mats were aragonite, calcite, and halite. Our analysis also revealed that the community structure of these mats is strongly influenced by salinity, redox potential, and temperature, indicating the significance of physicochemical micro-gradients in shaping microbial diversity. By characterizing the distribution, environmental characteristics, and microbial biodiversity of these mats, this work expands our understanding of microbial ecosystems in coastal habitats and highlights the importance of these complex systems for the health and functioning of coastal karst ecosystems.

Acknowledgments

We are grateful to the technicians from the Primary Productivity Laboratory from CINVESTAV-Mérida for their help during the field work. Thanks to Tania Cota and Anabel Suárez-Guevara for the contribution in the map and figures.

References

1. Stal LJ. Coastal microbial mats: the physiology of a small-scale ecosystem. South African Journal of Botany. 2001;67: 399–410. doi:10.1016/S0254-6299(15)31156-X

2. Stal LJ. Microbial mats in coastal environments. In: Stal LJ, Caumette P, editors. Microbial Mats. Berlin, Heidelberg: Springer Berlin Heidelberg; 1994. pp. 21–32. doi:10.1007/978-3-642-78991-5_2

3. Gerdes G. What are Microbial Mats? In: Seckbach J, Oren A, editors. Microbial Mats. Dordrecht: Springer Netherlands; 2010. pp. 3–25. doi:10.1007/978-90-481-3799-2_1

4. Hoffman P., Logan BW, Gebelein CD. Algal Mats, Cryptalgal Fabrics, and Structures, Hamelin Pool, Western Australia. AAPG Bulletin. 1972;56: 628–628. https://doi.org/10.1306/819A3F6A-16C5-11D7-8645000102C1865D

5. Play Ford PE, Cockbain AE. Chapter 8.2 Modern Algal Stromatolites at Hamelin Pool, A Hypersaline Barred Basin in Shark Bay, Western Australia. Developments in Sedimentology. Elsevier; 1976. pp. 389–411. doi:10.1016/S0070-4571(08)71147-6

6. Franks J, Stolz JF. Flat laminated microbial mat communities. Earth-Science Reviews. 2009;96: 163–172. doi:10.1016/j.earscirev.2008.10.004

7. Golubic S. Chapter 4.1 Organisms that Build Stromatolites. Developments in Sedimentology. Elsevier; 1976. pp. 113–126. doi:10.1016/S0070-4571(08)71132-4

8. Allen MA, Goh F, Burns BP, Neilan BA. Bacterial, archaeal and eukaryotic diversity of smooth and pustular microbial mat communities in the hypersaline lagoon of Shark Bay. Geobiology. 2009;7: 82–96. doi:10.1111/j.1472-4669.2008.00187.x

9. Skoog EJ, Moore KR, Gong J, Ciccarese D, Momper L, Cutts EM, et al. Metagenomic, (bio)chemical, and microscopic analyses reveal the potential for the cycling of sulfated EPS in Shark Bay pustular mats. ISME Communications. 2022;2: 43. doi:10.1038/s43705-022-00128-1

10. Wong HL, Smith D-L, Visscher PT, Burns BP. Niche differentiation of bacterial communities at a millimeter scale in Shark Bay microbial mats. Sci Rep. 2015;5: 15607. doi:10.1038/srep15607

11. Suosaari EP, Awramik SM, Reid RP, Stolz JF, Grey K. Living Dendrolitic Microbial Mats in Hamelin Pool, Shark Bay, Western Australia. Geosciences. 2018;8: 212. doi:10.3390/geosciences8060212

12. D'Agostino PM, Woodhouse JN, Liew HT, Sehnal L, Pickford R, Wong HL, et al. Bioinformatic, phylogenetic and chemical analysis of the UV-absorbing compounds scytonemin and mycosporine-like amino acids from the microbial mat communities of Shark Bay, Australia. Environmental Microbiology. 2019;21: 702–715. doi:10.1111/1462-2920.14517

13. Ford D, Williams PW. Karst hydrogeology and geomorphology. Rev. ed. Chichester, England; a Hoboken, NJ: John Wiley & Sons; 2007.

14. Bauer-Gottwein P, Gondwe BRN, Charvet G, Marín LE, Rebolledo-Vieyra M, Merediz-Alonso G. Review: The Yucatán Peninsula karst aquifer, Mexico. Hydrogeol J. 2011;19: 507–524. doi:10.1007/s10040-010-0699-5

15. Herrera-Silveira JA, Ramírez-Ramírez J. Salinity and nutrients in the coastal lagoons of Yucatan, Mexico. SIL Proceedings, 1922-2010. 1998;26: 1473–1478. doi:10.1080/03680770.1995.11900971

16. Ortiz-Milan S. Project of Recovery the Biological Conditions of the Production System in Saltworks of Industria Salinera de Yucatan S.A. de C.V. (ISYSA) Damaged by the Hurricane Isidore in September of 2002. Global NEST Journal. 2013;11: 91–95. doi:10.30955/gnj.000568

17. McKee KL, Mendelssohn IA, Hester MW. Reexamination of pore water sulfide concentrations and redox potentials near the aerial roots of Rhizophora mangle and Avicennia germinans. American J of Botany. 1988;75: 1352–1359. doi:10.1002/j.1537-2197.1988.tb14196.x

18. Parada AE, Needham DM, Fuhrman JA. Every base matters: assessing small subunit RRNA primers for marine microbiomes with mock communities, time series and global field samples. Environmental Microbiology. 2016;18: 1403–1414. doi:10.1111/1462-2920.13023

19. Needham D, Fichot E, Parada A, Yeh Y-C, Fuhrman J. Fuhrman Lab 515F-926R 16S and 18S rRNA Sequencing Protocol v1. 2018. doi:10.17504/protocols.io.nkhdct6

20. Caporaso JG, Kuczynski J, Stombaugh J, Bittinger K, Bushman FD, Costello EK, et al. QIIME allows analysis of high-throughput community sequencing data. Nat Methods. 2010;7: 335–336. doi:10.1038/nmeth.f.303

21. Callahan BJ, McMurdie PJ, Rosen MJ, Han AW, Johnson AJA, Holmes SP. DADA2: High-resolution sample inference from Illumina amplicon data. Nat Methods. 2016;13: 581–583. doi:10.1038/nmeth.3869

22. Rognes T, Flouri T, Nichols B, Quince C, Mahé F. VSEARCH: a versatile open source tool for metagenomics. PeerJ. 2016;4: e2584. doi:10.7717/peerj.2584

23. Katoh K, Standley DM. MAFFT Multiple Sequence Alignment Software Version 7: Improvements in Performance and Usability. Molecular Biology and Evolution. 2013;30: 772–780. doi:10.1093/molbev/mst010

24. Price MN, Dehal PS, Arkin AP. FastTree 2 – Approximately Maximum-Likelihood Trees for Large Alignments. Poon AFY, editor. PLoS ONE. 2010;5: e9490. doi:10.1371/journal.pone.0009490

25. McMurdie PJ, Holmes S. phyloseq: An R Package for Reproducible Interactive Analysis and Graphics of Microbiome Census Data. Watson M, editor. PLoS ONE. 2013;8: e61217. doi:10.1371/journal.pone.0061217

26. Wickham H. ggplot2: Elegant Graphics for Data Analysis. New York, NY: Springer New York; 2009. doi:10.1007/978-0-387-98141-3

27. Segata N, Izard J, Waldron L, Gevers D, Miropolsky L, Garrett WS, et al. Metagenomic biomarker discovery and explanation. Genome Biol. 2011;12: R60. doi:10.1186/gb-2011-12-6-r60

28. The Galaxy Community, Afgan E, Nekrutenko A, Grüning BA, Blankenberg D, Goecks J, et al. The Galaxy platform for accessible, reproducible and collaborative biomedical analyses: 2022 update. Nucleic Acids Research. 2022;50: W345–W351. doi:10.1093/nar/gkac247

29. Yanez-Montalvo A, Gómez-Acata S, Águila B, Hernández-Arana H, Falcón LI. The microbiome of modern microbialites in Bacalar Lagoon, Mexico. Senko JM, editor. PLoS ONE. 2020;15: e0230071. doi:10.1371/journal.pone.0230071

30. Yanez-Montalvo A, Águila B, Gómez-Acata S, Mass-Vargas M, Cabanillas-Terán N, Vega-Zepeda A, et al. Depth Related Structure and Microbial Composition of Microbialites in a Karst Sinkhole, Cenote Azul, Mexico. Geomicrobiology Journal. 2021;38: 237–251. doi:10.1080/01490451.2020.1836086

31. Schmitter-Soto JJ, Comín FA, Escobar-Briones E, Herrera-Silveira J, Alcocer J, Suárez-Morales E, et al. Hydrogeochemical and biological characteristics of cenotes in the Yucatan Peninsula (SE Mexico). In: Alcocer J, Sarma SSS, editors. Advances in Mexican Limnology: Basic and Applied Aspects. Dordrecht: Springer Netherlands; 2002. pp. 215–228. doi:10.1007/978-94-010-0415-2_19

32. Schaefer B, Grice K, Coolen MJL, Summons RE, Cui X, Bauersachs T, et al. Microbial life in the nascent Chicxulub crater. Geology. 2020;48: 328–332. doi:10.1130/G46799.1

33. John J, Hay M, Paton J. Cyanobacteria in benthic microbial communities in coastal salt lakes in Western Australia. algol_stud. 2009;130: 125–135. doi:10.1127/1864-1318/2009/0130-0125

34. Lacap DC, Barraquio W, Pointing SB. Thermophilic microbial mats in a tropical geothermal location display pronounced seasonal changes but appear resilient to stochastic disturbance. Environmental Microbiology. 2007;9: 3065–3076. doi:10.1111/j.1462-2920.2007.01417.x

35. Lozupone CA, Knight R. Global patterns in bacterial diversity. Proc Natl Acad Sci USA. 2007;104: 11436–11440. doi:10.1073/pnas.0611525104

36. Hicks N, Liu X, Gregory R, Kenny J, Lucaci A, Lenzi L, et al. Temperature Driven Changes in Benthic Bacterial Diversity Influences Biogeochemical Cycling in Coastal Sediments. Front Microbiol. 2018;9: 1730. doi:10.3389/fmicb.2018.01730

37. Edlund A, Hårdeman F, Jansson JK, Sjöling S. Active bacterial community structure along vertical redox gradients in Baltic Sea sediment. Environmental Microbiology. 2008;10: 2051–2063. doi:10.1111/j.1462-2920.2008.01624.x

38. Lugomela C, Bergman B. Biological N 2 -fixation on Mangrove Pneumatophores: Preliminary Observations and Perspectives. AMBIO: A Journal of the Human Environment. 2002;31: 612–613. doi:10.1579/0044-7447-31.7.612

39. McKee KL. Biophysical controls on accretion and elevation change in Caribbean mangrove ecosystems. Estuarine, Coastal and Shelf Science. 2011;91: 475–483. doi:10.1016/j.ecss.2010.05.001

40. Lee R, Joye S. Seasonal patterns of nitrogen fixation and denitrification in oceanic mangrove habitats. Mar Ecol Prog Ser. 2006;307: 127–141. doi:10.3354/meps307127

41. Gontharet S, Crémière A, Blanc-Valleron M-M, Sebilo M, Gros O, Laverman AM, et al. Sediment characteristics and microbial mats in a marine mangrove, Manche-à-eau lagoon (Guadeloupe). J Soils Sediments. 2017;17: 1999–2010. doi:10.1007/s11368-016-1555-6

42. Holguin G, Vazquez P, Bashan Y. The role of sediment microorganisms in the productivity, conservation, and rehabilitation of mangrove ecosystems: an overview. Biology and Fertility of Soils. 2001;33: 265–278. doi:10.1007/s003740000319

43. Kirk Harris J, Gregory Caporaso J, Walker JJ, Spear JR, Gold NJ, Robertson CE, et al. Phylogenetic stratigraphy in the Guerrero Negro hypersaline microbial mat. The ISME Journal. 2013;7: 50–60. doi:10.1038/ismej.2012.79

44. D'haeseleer P, Lee JZ, Prufert-Bebout L, Burow LC, Detweiler AM, Weber PK, et al. Metagenomic analysis of intertidal hypersaline microbial mats from Elkhorn Slough, California, grown with and without molybdate. Stand in Genomic Sci. 2017;12: 67. doi:10.1186/s40793-017-0279-6

45. Louca S, Polz MF, Mazel F, Albright MBN, Huber JA, O'Connor MI, et al. Function and functional redundancy in microbial systems. Nat Ecol Evol. 2018;2: 936–943. doi:10.1038/s41559-018-0519-1

46. Uritskiy G, Getsin S, Munn A, Gomez-Silva B, Davila A, Glass B, et al. Halophilic microbial community compositional shift after a rare rainfall in the Atacama Desert. The ISME Journal. 2019;13: 2737–2749. doi:10.1038/s41396-019-0468-y

47. Klappenbach JA, Pierson BK. Phylogenetic and physiological characterization of a filamentous anoxygenic photoautotrophic bacterium ?Candidatus Chlorothrix halophila? gen. nov., sp. nov., recovered from hypersaline microbial mats. Archives of Microbiology. 2004;181: 17–25. doi:10.1007/s00203-003-0615-7

48. Fukunaga Y, Kurahashi M, Sakiyama Y, Ohuchi M, Yokota A, Harayama S. Phycisphaera mikurensis gen. nov., sp. nov., isolated from a marine alga, and proposal of Phycisphaeraceae fam. nov., Phycisphaerales ord. nov. and Phycisphaerae classis nov. in the phylum Planctomycetes. J Gen Appl Microbiol. 2009;55: 267–275. doi:10.2323/jgam.55.267

49. Rios-Del Toro EE, Valenzuela EI, Ramírez JE, López-Lozano NE, Cervantes FJ. Anaerobic Ammonium Oxidation Linked to Microbial Reduction of Natural Organic Matter in Marine Sediments. Environ Sci Technol Lett. 2018;5: 571–577. doi:10.1021/acs.estlett.8b00330

50. Singh S, Keating C, Ijaz UZ, Hassard F. Molecular insights informing factors affecting low temperature anaerobic applications: Diversity, collated core microbiomes and complexity stability relationships in LCFA-fed systems. Science of The Total Environment. 2023;874: 162420. doi:10.1016/j.scitotenv.2023.162420

51. Ward LM, Lingappa UF, Grotzinger JP, Fischer WW. Microbial mats in the Turks and Caicos Islands reveal diversity and evolution of phototrophy in the Chloroflexota order Aggregatilineales. Environmental Microbiome. 2020;15: 9. doi:10.1186/s40793-020-00357-8

52. Lei X, Li Y, Wang G, Chen Y, Lai Q, Chen Z, et al. Phaeodactylibacter luteus sp. nov., isolated from the oleaginous microalga Picochlorum sp. International Journal of Systematic and Evolutionary Microbiology. 2015;65: 2666–2670. doi:10.1099/ijs.0.000321

53. Kawaichi S, Ito N, Kamikawa R, Sugawara T, Yoshida T, Sako Y. Ardenticatena maritima gen. nov., sp. nov., a ferric iron- and nitrate-reducing bacterium of the phylum ' Chloroflexi ' isolated from an iron-rich coastal hydrothermal field, and description of Ardenticatenia classis nov. International Journal of Systematic and Evolutionary Microbiology. 2013;63: 2992–3002. doi:10.1099/ijs.0.046532-0

54. Mongodin EF, Nelson KE, Daugherty S, DeBoy RT, Wister J, Khouri H, et al. The genome of Salinibacter ruber : Convergence and gene exchange among hyperhalophilic bacteria and archaea. Proc Natl Acad Sci USA. 2005;102: 18147–18152. doi:10.1073/pnas.0509073102

55. Mongodin EF, Nelson KE, Daugherty S, DeBoy RT, Wister J, Khouri H, et al. The genome of Salinibacter ruber : Convergence and gene exchange among hyperhalophilic bacteria and archaea. Proc Natl Acad Sci USA. 2005;102: 18147–18152. doi:10.1073/pnas.0509073102

56. Muyzer G, Sorokin DY, Mavromatis K, Lapidus A, Clum A, Ivanova N, et al. Complete genome sequence of Thioalkalivibrio "sulfidophilus" HL-EbGr7. Stand Genomic Sci. 2011;4: 23–35. doi:10.4056/sigs.1483693

57. Oh H-M, Giovannoni SJ, Lee K, Ferriera S, Johnson J, Cho J-C. Complete Genome Sequence of Robiginitalea biformata HTCC2501. J Bacteriol. 2009;191: 7144–7145. doi:10.1128/JB.01191-09

58. Sun L, Toyonaga M, Ohashi A, Tourlousse DM, Matsuura N, Meng X-Y, et al. Lentimicrobium saccharophilum gen. nov., sp. nov., a strictly anaerobic bacterium representing a new family in the phylum Bacteroidetes, and proposal of Lentimicrobiaceae fam. nov. International Journal of Systematic and Evolutionary Microbiology. 2016;66: 2635–2642. doi:10.1099/ijsem.0.001103

Supporting information

S1 Table. Mineral composition of microbial mats. Comparative table of the mineral composition of studied microbial mats from the Yucatan Peninsula. (x) presence, (-) absence.

S2 Table. 16s rRNA gene sequencing stats. Sequencing stats before and after denoising and chimera remotion.

S3 Table. Observed microbial diversity. Classified ASV's from 16s rRNA gene sequencing of microbial mats.

 Click here to access/download;Figure;Figure1 (1)[1].tif

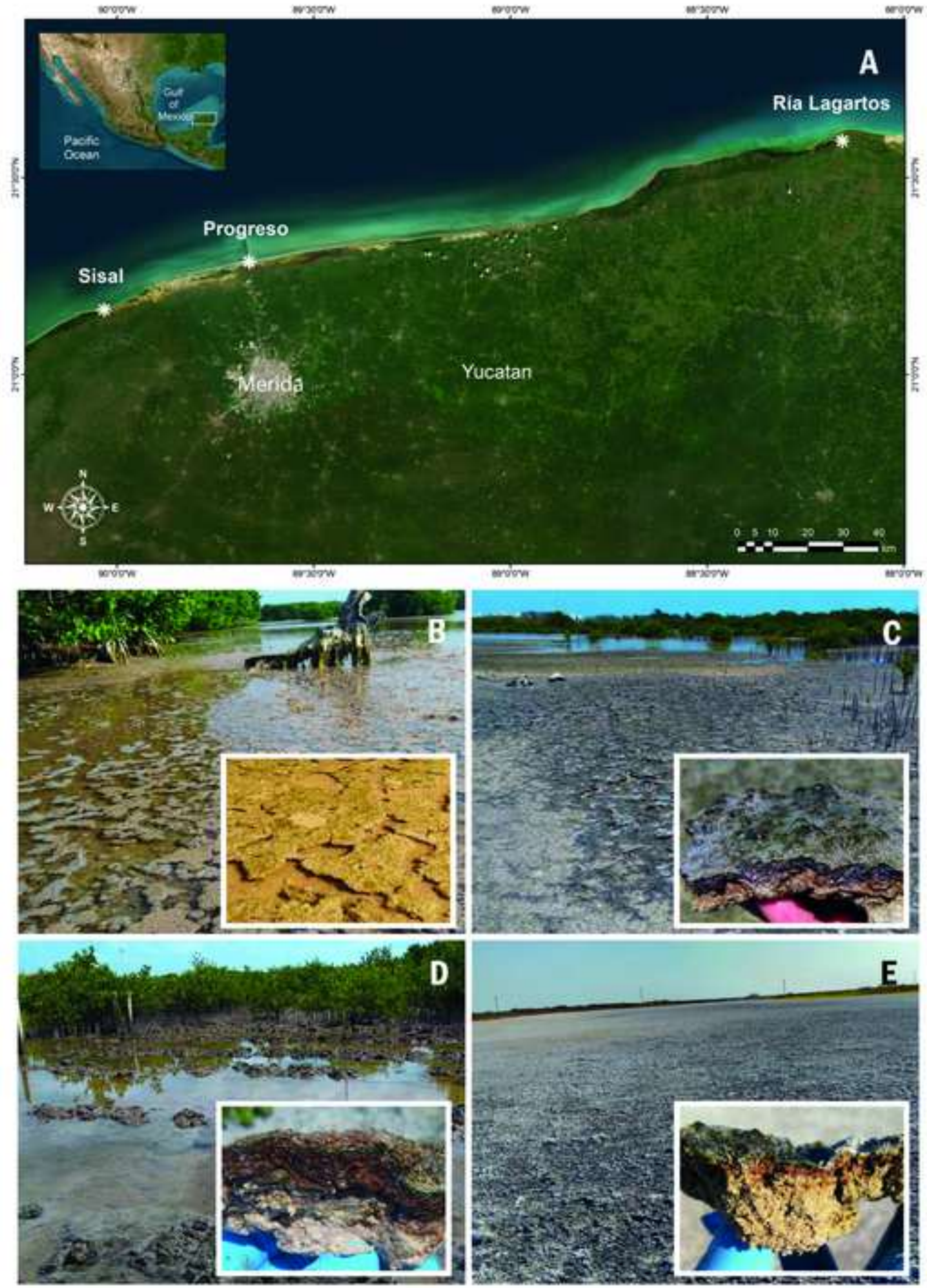

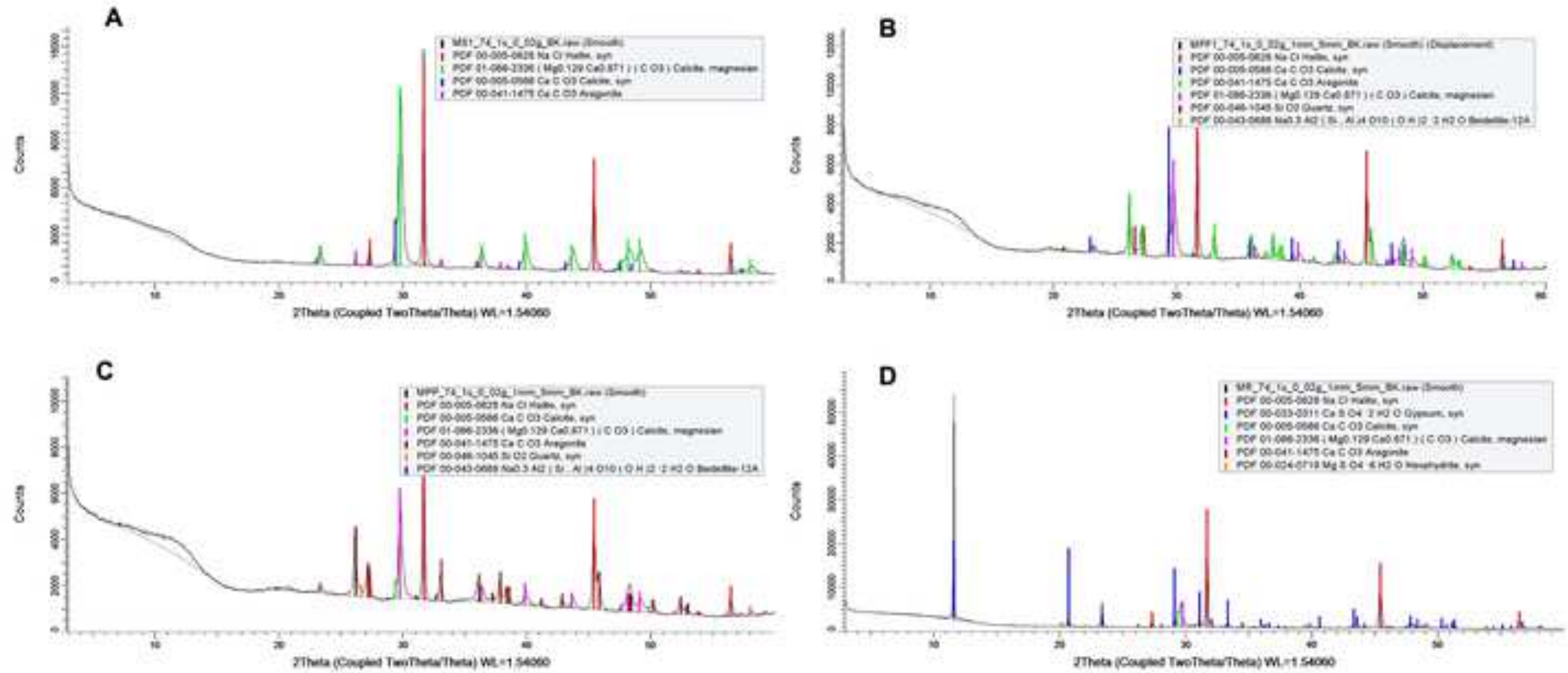
A
B
C
D
Counts
2Theta (Coupled TwoTheta/Theta) WL=1.54060

Figure

Click here to access/download;Figure;Figure3 (1).tif

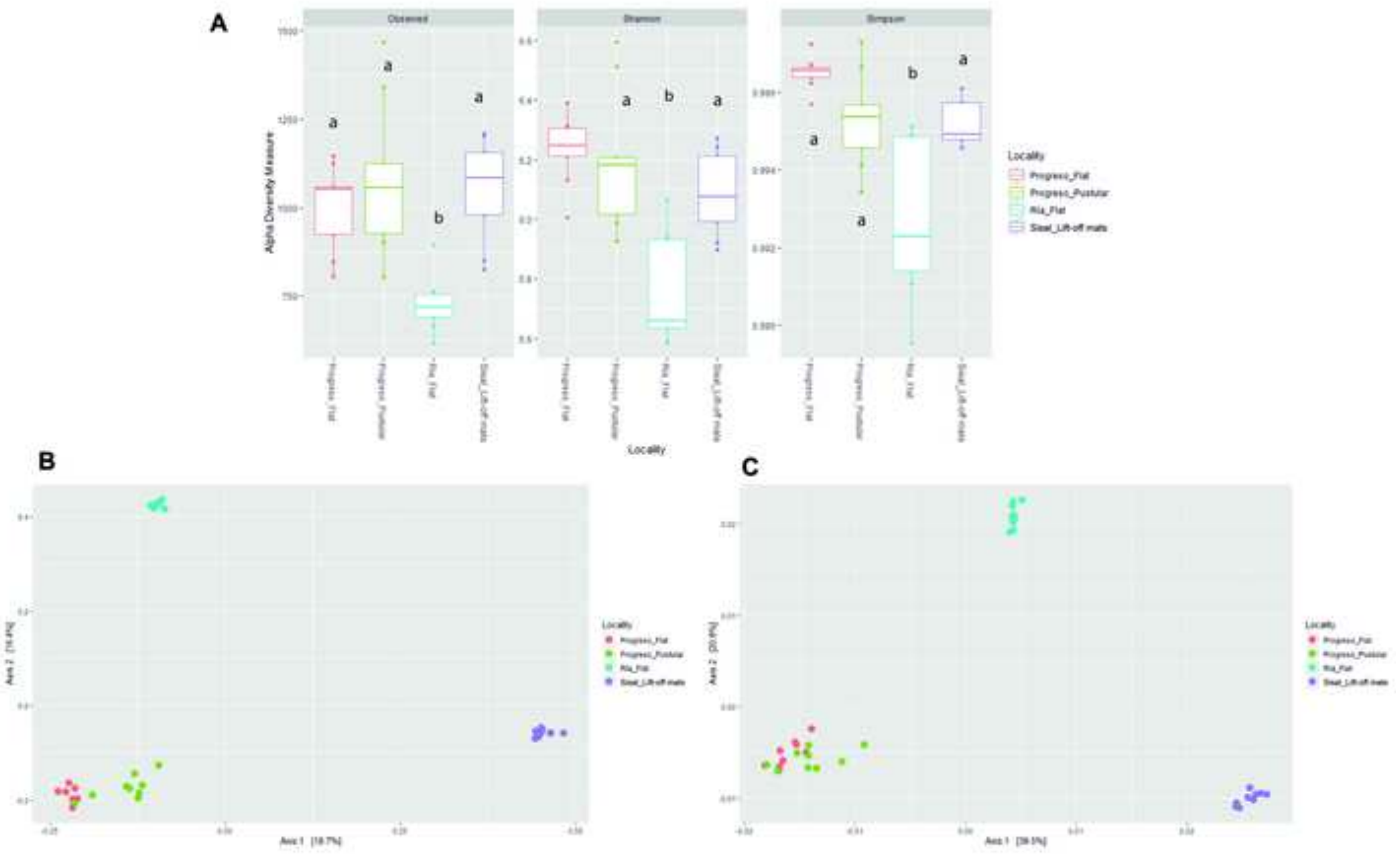

Figure Click here to access/download;Figure;Figure4 (1).tif

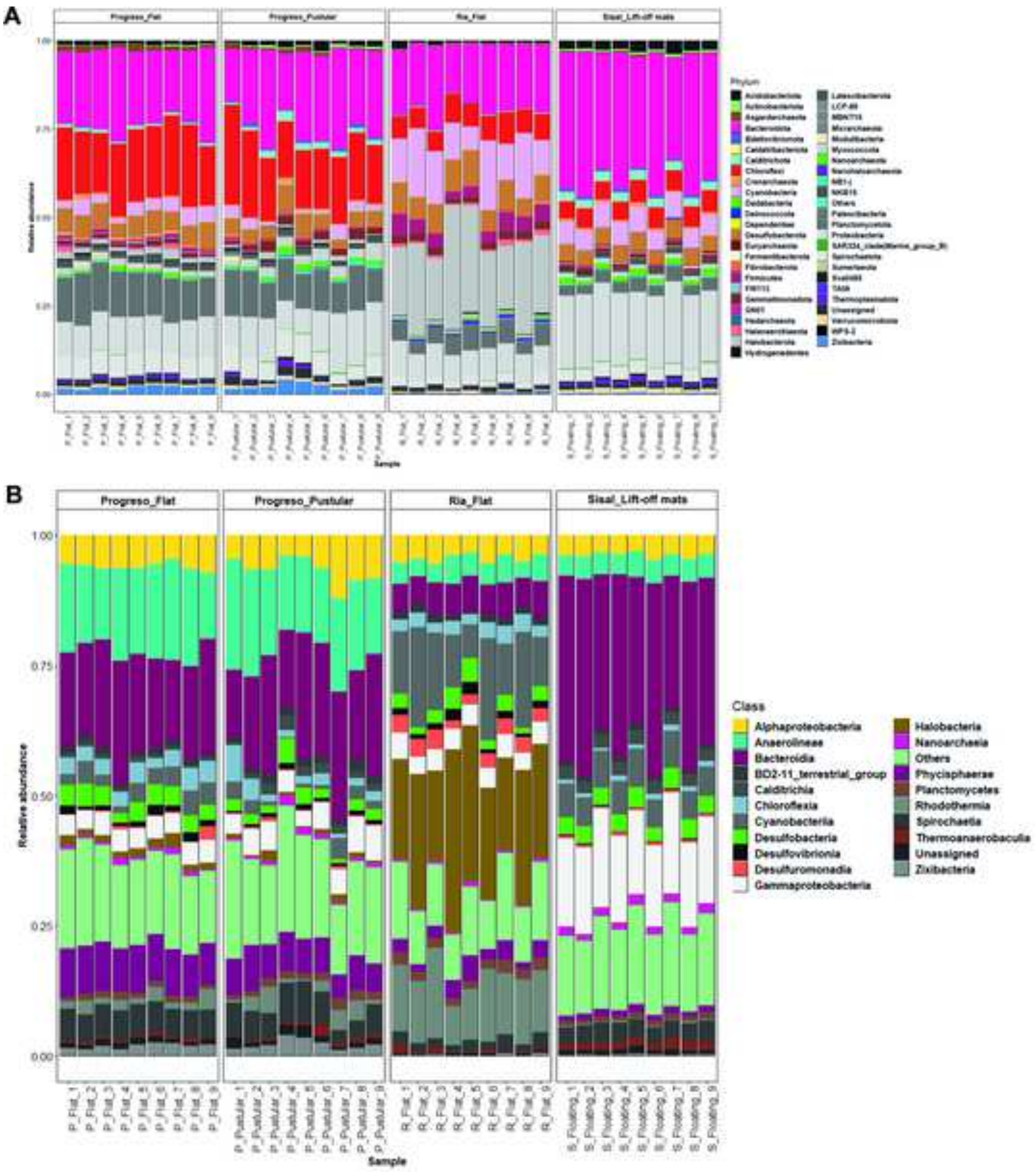

 Click here to access/download;Figure;Figure5 (1).tif

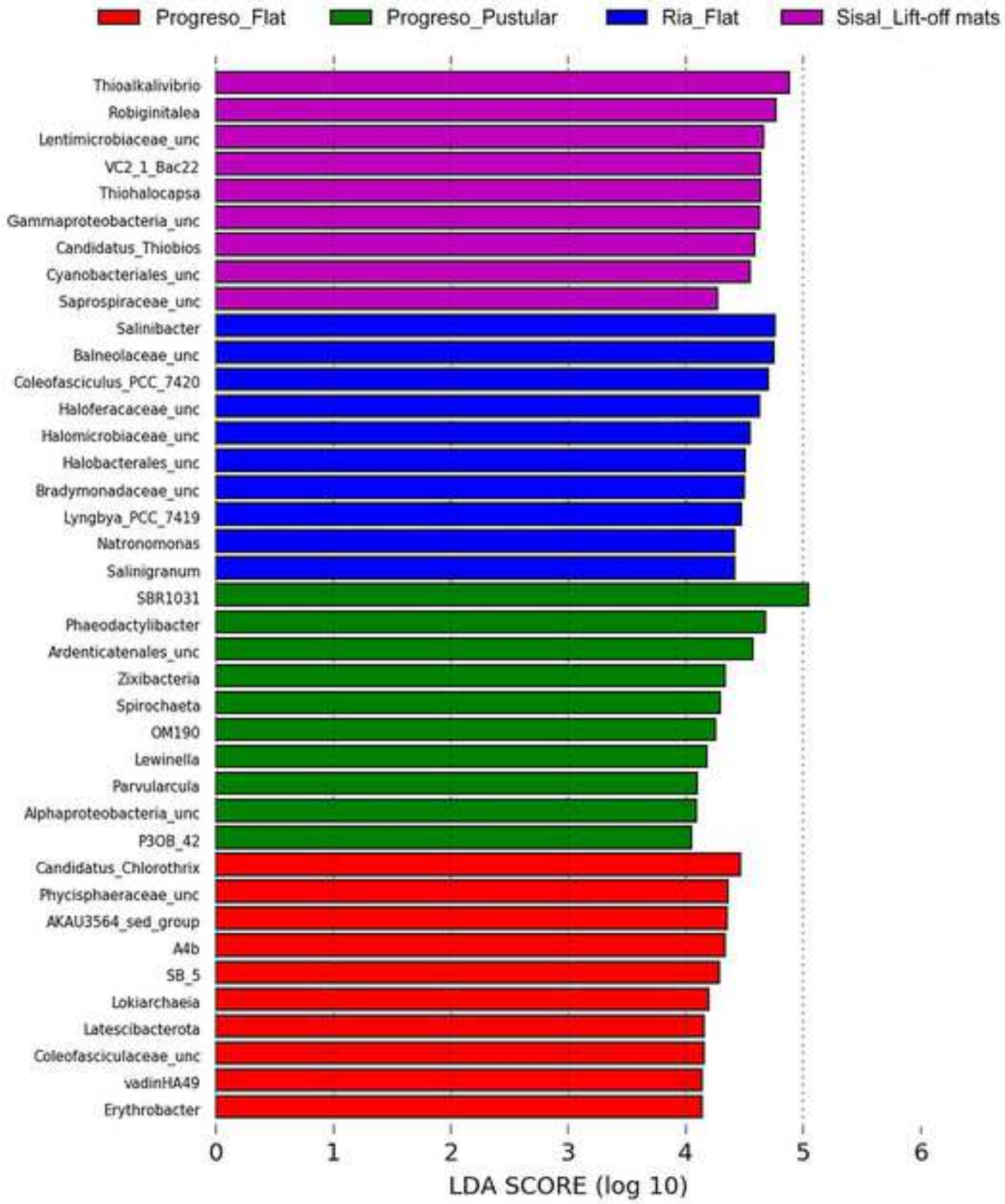

Click here to access/download
Supporting Information
Supplementary Table S1.docx

Click here to access/download
Supporting Information
Supplementary Table S2.xlsx

Click here to access/download
Supporting Information
Supplementary Table S3.xlsx
